# The Role of Key Amino Acids of the Human Fe(II)/2OG-Dependent Dioxygenase ALKBH3 in Structural Dynamics and Repair Activity toward Methylated DNA

**DOI:** 10.3390/ijms25021145

**Published:** 2024-01-17

**Authors:** Lyubov Yu. Kanazhevskaya, Alexey A. Gorbunov, Maria V. Lukina, Denis A. Smyshliaev, Polina V. Zhdanova, Alexander A. Lomzov, Vladimir V. Koval

**Affiliations:** 1 Institute of Chemical Biology and Fundamental Medicine (ICBFM), 8 Lavrentiev Ave., Novosibirsk 630090, Russia; 2 Department of Natural Sciences, Novosibirsk State University, 1 Pirogova St., Novosibirsk 630090, Russia

**Keywords:** DNA repair, DNA methylation, dioxygenase ALKBH3, site-directed mutagenesis, circular dichroism, stopped-flow method, conformational dynamics, fluorescent spectroscopy

## Abstract

Non-heme dioxygenases of the AlkB family hold a unique position among enzymes that repair alkyl lesions in nucleic acids. These enzymes activate the Fe(II) ion and molecular oxygen through the coupled decarboxylation of the 2-oxoglutarate co-substrate to subsequently oxidize the substrate. ALKBH3 is a human homolog of *E. coli* AlkB, which displays a specific activity toward N1-methyladenine and N3-methylcytosine bases in single-stranded DNA. Due to the lack of a DNA-bound structure of ALKBH3, the basis of its substrate specificity and structure–function relationships requires further exploration. Here we have combined biochemical and biophysical approaches with site-directed mutational analysis to elucidate the role of key amino acids in maintaining the secondary structure and catalytic activity of ALKBH3. Using stopped-flow fluorescence spectroscopy we have shown that conformational dynamics play a crucial role in the catalytic repair process catalyzed by ALKBH3. A transient kinetic mechanism, which comprises the steps of the specific substrate binding, eversion, and anchoring within the DNA-binding cleft, has been described quantitatively by rate and equilibrium constants. Through CD spectroscopy, we demonstrated that replacing side chains of Tyr143, Leu177, and His191 with alanine results in significant alterations in the secondary structure content of ALKBH3 and decreases the stability of mutant proteins. The bulky side chain of Tyr143 is critical for binding the methylated base and stabilizing its flipped-out conformation, while its hydroxyl group is likely involved in facilitating the product release. The removal of the Leu177 and His191 side chains substantially affects the secondary structure content and conformational flexibility, leading to the complete inactivation of the protein. The mutants lacking enzymatic activity exhibit a marked decrease in antiparallel β-strands, offset by an increase in the helical component.

## 1. Introduction

Fe(II)/2-oxoglutarate–dependent dioxygenases form a diverse superfamily of proteins, which use a non-heme iron center to catalyze an oxidative decarboxylation [1]. The enzymatic process requires oxidation of a co-substrate 2-oxoglutarate (2OG) for activation of molecular oxygen and subsequent formation of a highly reactive Fe(IV)-oxo species [2]. Fe(II)/2OG-dependent dioxygenases involved in DNA/RNA repair and protein modification belong to the AlkB subfamily [3]. In mammalian cells, the AlkB-like dioxygenases are represented by nine homologs with distinct functions (ALKBH1-ALKBH8 and FTO). In particular, human AlkB homologs 2 and 3 (ALKBH2, ALKBH3) have been proven to be repair proteins, which directly remove alkyl and etheno groups from nucleic acid bases [4,5]. ALKBH3 and AlkB have more in common regarding the substrate specificity than do ALKBH3 and ALKBH2, and they can oxidize the methyl group of N1-methyladenine (m1A), N3-methylcytosine (m3C), and N1-methylguanine (m1G) predominantly in single-stranded (ss) DNA or RNA. In contrast to ALKBH2, ALKBH3 repairs m3C more effectively than m1A, whereas AlkB acts equally well against these lesions [6,7]. Although the biological function of ALKBH3 is not completely understood, some data indicate its demethylation activity on ssDNA regions arising during replication or transcription, as well as on m1A-containing mRNA in vivo [8,9]. For example, it has been shown that the direct protein–protein interaction of ALKBH3 with the RAD51C recombinase enhances the ALKBH3-mediated demethylation rate and efficiency for the 3′-tailed m3C-DNA substrate [10]. Furthermore, in vivo experiments have established that such stimulation is critically important for repair of genomic DNA during homologous recombination. Numerous links between altered ALKBH3 expression and human cancers make this protein a potential therapeutic target in tumorigenesis [11,12,13].

**Figure 1:**
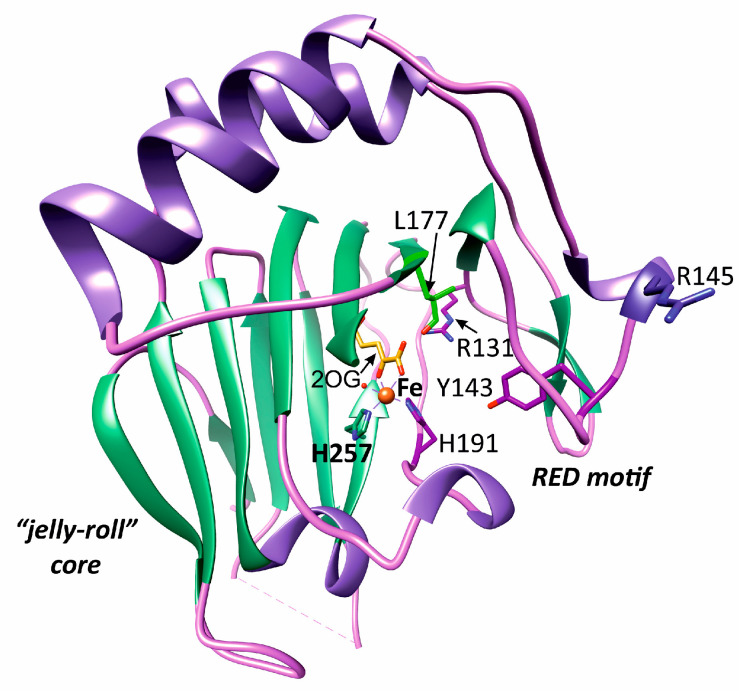
The structure of the ALKBH3 protein with Fe (brown) and 2OG (yellow) (PDB ID: 2IUW) [14]. Key coordinating amino acid residues are indicated. Α-helixes and turns are colored in violet, and β-strands are colored in green.

The structure of ALKBH3 possess all the characteristic domains typical for AlkB-like proteins: a β-strand “jellyroll” core (double-stranded β-helix domain, DSBH), a DNA/RNA recognition “lid”, and a poorly ordered N-terminal extension (Figure 1) [14]. A highly conserved His^1^-X-Asp/Glu-X_n_-His^2^ motif, coordinating a catalytically active iron center within the DSBH motif, consists of His191, Asp193, and His257 residues. The substrate-binding pocket of ALKBH3 is considerably more polar and positively charged compared to the hydrophobic active site of AlkB. High-resolution crystal structures have revealed that the topology of the substrate recognition region of ALKBH3 is more similar to that of ALKBH2, which utilizes polar amino acids to provide selection and stabilization of the alkylated base [14,15,16]. It is considered that the substrate specificity of AlkB-like proteins depends on distinctive loop regions adapted to bind and stabilize ss or dsDNA [17]. By means of these loops, the *E. coli* AlkB protein can squeeze a damaged strand of the ss or ds substrate so that a methylated base is pushed out and placed in the active site cavity. ALKBH2 inserts its loops into dsDNA grooves to carry out dealkylation. In turn, ALKBH3 possesses a shallower binding cleft and shorter loop motifs suited to act on ssDNA or RNA. However, the lengths and amino acid composition of the hairpins are not fully consistent between the ALKBH2 and ALKBH3 proteins. Structural comparison of the key DNA-binding elements of ALKBH3 and its homologs is impeded by the absence of a DNA-bound ALKBH3 structure. Moreover, the crystal structure of the truncated version (ΔN69) of the free protein (PDB 2IUW) lacks visible electron density in the important loop region between β10 and β11. A combined cross-linking and motif-swapping study has shown the β-hairpin motif of a strand determination loop (a.a. 98–107) of ALKBH2 to contain a hydrophobic VFG triad (Val101, Phe102, Gly103), whereas ALKBH3 contains a hydrophilic RED triad (Arg122, Glu123, Asp124) in the corresponding location (a.a. 118–129) (Figure 1). This distinction allows ALKBH2 to discriminate and capture unstable base pairing within the double helix, while ALKBH3 is prevented from binding dsDNA [18,19]. Nevertheless, amino acids of the ALKBH3’s antiparallel β-hairpin, including the RED motif, may intercalate the DNA strand near the damage site and partially occupy the space of a flipped-out base. On the other hand, it remains poorly elucidated to date which residues complement the function of RED at the opposite edge of the DNA-binding cleft in order to fix the damaged base within the active site. Among other possibilities, Arg131, Tyr143, and Arg145 have been proposed as interacting with the substrate base in the course of a productive complex formation [14,18]. It is clear that further investigations of ALKBH3-DNA interactions using diverse analytical methods are required for a deeper understanding of the enzyme–substrate interaction mechanism.

The efficient interaction of the ALKBH3 protein with methylated DNA is only achieved through proper coordination between key amino acid residues and their nucleotide counterparts. Removal or replacement of the side chains of conservative amino acids generally leads to a weakening or complete loss of demethylation activity [14]. In the present study, we attempted to investigate how the substitution of amino acids coordinating the DNA substrate, Fe(II), and 2OG affect the structure, dynamic properties, and progression of individual steps of the ALKBH3 catalytic cycle. Additionally, we explored the role of the presumably significant residue Tyr143 in DNA substrate coordination and catalysis. To achieve these objectives, site-directed mutagenesis of three conservative residues, namely Tyr143, Leu177, and His191, was performed. The side chain of Tyr143 may be involved in coordination of the damage similar to residue Asp135 in AlkB that forms a hydrogen bond to the N6 of m1A [14]. We have chosen Tyr143 for analysis because it has not previously been studied. Unlike Arg145, Tyr143 is conservative and directed inward toward the DNA-binding cleft in the crystal structure (Figure 1). Here, we sequentially replaced Tyr143 with Phe or Ala to remove its side chain of the polar OH moiety or to completely eliminate the bulky residue, respectively. Leu177 was chosen for mutagenesis as it is buried inside the active site pocket and may form an indirect contact with the co-substrate 2OG. It has been suggested earlier that, in the absence of the specific substrate, the side chain of Leu177 may undergo oxidation by the hydroxyl radical generated by uncoupled 2OG decarboxylation [14]. His191 belongs to the conservative triad coordinating the Fe(II) ion, so its replacement with Ala prevents the enzyme from being catalytically active and may lead to a significant distortion of the active site architecture.

Spectroscopic methods have been widely applied to study the 2OG dioxygenases involved in nucleic acid repair/modification due to their capabilities to capture intermediate species and conformational transitions of the enzyme–substrate complex, as well as to explore ligand binding under near-physiological conditions [20]. For example, NMR, time-resolved fluorescence spectroscopy, and circular dichroism (CD) have demonstrated a dynamic behavior of AlkB conformation that accompanies the binding of cofactors and a DNA substrate [21,22]. Using a stopped-flow (SF) approach combined with fluorescence spectroscopy we previously demonstrated how the conformational dynamics of AlkB and ALKBH2 proteins induce and follow the recognition and processing of the alkylated substrate [7,23]. The present work was performed by using biochemical and biophysical approaches. CD spectroscopy was employed to address the potential effect of selected amino acid mutation on ALKBH3’s secondary structure content and thermal stability. Our data indicated a significant decrease in antiparallel β-strands for catalytically inactive mutants, which correlated with an increase in α-helix and turn content. The removal of the side chains of Tyr143, Leu177, and His191 reduced ALKBH3’s ability to bind cofactors and DNA, suggesting a significant change in the geometry of the DNA-binding pocket. The ability of wild-type (wt) and mutant ALKBH3 proteins to bind ligands and damaged DNA was characterized using equilibrium fluorescence titration. The SF method with Förster resonance energy transfer (FRET) detection has revealed the dynamic behavior of the ALKBH3-DNA complex during recognition and oxidation of m3C-containing DNA. Conformational transitions accompanying the catalytic cycle described a five-step molecular mechanism and kinetic parameters were determined. 

## 2. Results

### 2.1. Enzymatic Activity of the Wild-Type and Mutant ALKBH3 Proteins

**Figure 2:**
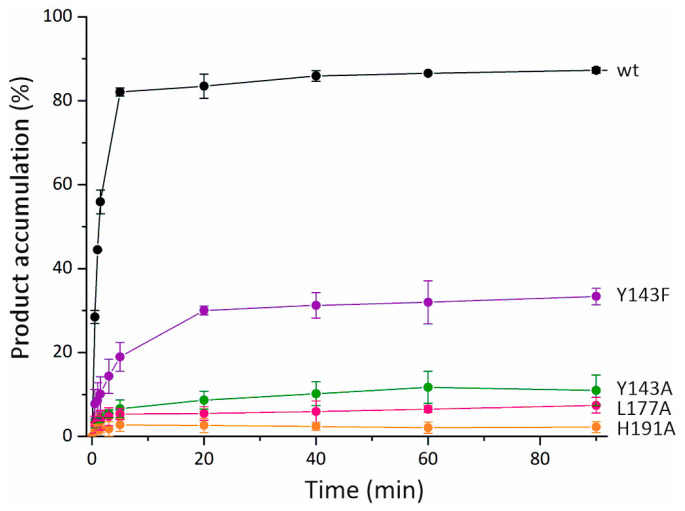
The catalytic activity of wt and mutant ALKBH3 proteins on single-stranded DNA substrate m3C_FAM. Time courses of the dealkylated product accumulation were obtained at 25 °C by mixing an equimolar amount (2 μM) of enzyme and FAM-labeled DNA substrate dissolved in the reaction buffer containing 40 μM Fe(II) and 1 mM 2OG. Each reaction time point is the mean ± SD of three technical replicates.

The enzymatic activity of wt ALKBH3 dioxygenase and the Y143F, Y143A, L177A, and H191A mutants was investigated in a reaction with a 15nt single-stranded oligonucleotide substrate containing m3C at the ninth position from the 5’-end (see Section 4). The resultant kinetic profiles of product accumulation over time showed a high level of demethylating activity of the wild-type enzyme, consistent with the literature [5], while the activity of the mutants was reduced to varying degrees (Figure 2, Supplementary Figure S1). Specifically, the substitution of Tyr143 with Phe resulted in a maximum DNA substrate demethylation degree of 30% over a 90 min reaction period, whereas replacement with Ala decreased the product yield to 10%. For the L177A mutation, product accumulation did not surpass 7%, and for the H191A substitution, product formation that reliably exceeded the measurement error was undetectable.

The data on enzymatic activity indicate that removing the polar hydroxyl group from Tyr143 leads to a fourfold reduction in the demethylation capacity of ALKBH3 but does not completely inhibit its activity. Conversely, the complete removal of the bulky moiety in the Y143A substitution virtually eliminates the enzyme’s activity. Therefore, the correct positioning of the damaged nucleotide within the active site pocket is not only ensured by polar contacts between the hydroxyl group of tyrosine and the amino group of m3C but also by the presence of a large hydrophobic substituent. It appears that the replacement of the Tyr143 side chain with Ala leads to significant disruption in ALKBH3’s ability to interact specifically with the lesion. As anticipated, substituting the side chains of Leu177 and His191 with Ala nearly abolished the demethylating ability of these mutants. However, it remained to be elucidated which specific disruptions in the L177A and H191A structure are critical for coordinating the co-substrate 2OG molecule and the cofactor Fe(II). 

The curves representing the accumulation of the product of m3C-containing ssDNA oxydation by the wt and Y143F ALKBH3 proteins were quantitatively analyzed using a single exponential fit (see Section 4). This analysis yielded the observed rate constants for demethylation, *k_obs_*, which were found to be 0.70 ± 0.08 min^−1^ and 0.17 ± 0.02 min^−1^ for the wt and Y143F, respectively. Previously, under identical conditions, the *k_obs_* values for another human dioxygenase, ALKBH2, were determined to be 0.22 min^−1^ for a similar m3C-containing substrate [7]. This difference in rate constants suggests that ALKBH3 exhibits a higher specificity for m3C damage compared to its homologue ALKBH2.

### 2.2. Conformational Dynamics of the ALKBH3-DNA Complex Revealed by SF Fluorescence Spectroscopy

**Figure 3:**
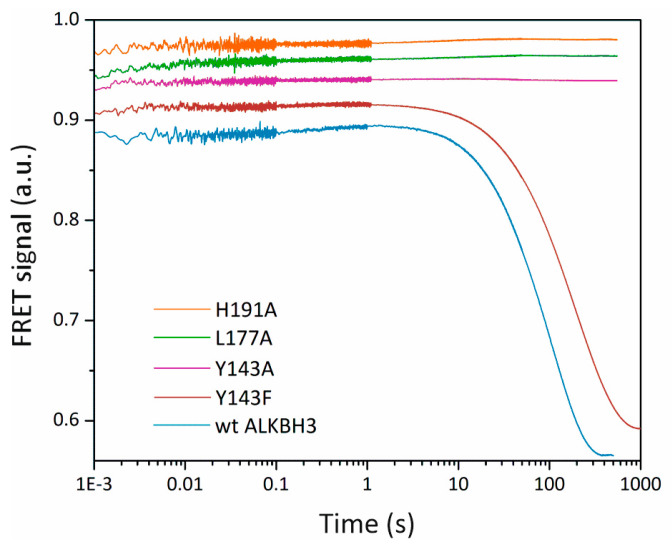
Time courses of the FRET signal generated by the FAM/BHQ1 pair under interactions of wt, Y143F, Y143A, L177A, and H191A ALKBH3 with m3C-containing DNA. The fluorescence traces represent changes in the FRET signal after rapid mixing of 1.5 µM m3C_FRET substrate with 1.5 µM protein in the presence of 40 µM Fe(II) and 1 mM 2OG. Each curve was collected in a split mode within four time intervals (up to 0.1, 1, 50, and 500 or 1000 s) in 4–5 replicates.

In previous research, we employed a stopped-flow (SF) method to detect conformational changes in the dioxygenases AlkB and ALKBH2, as well as their substrates, throughout the catalytic cycle [7,23]. The SF technique provides excellent opportunities for studying the conformational dynamics of protein–nucleic acid complexes under pre-steady-state conditions within the millisecond time range. Conformational transitions are typically detected by measuring the fluorescent signal from the tryptophan residues of the enzyme or fluorescent labels incorporated into the DNA substrate sequence. In the case of ALKBH3, SF experiments revealed that the dye pair 5’-FAM/3’-BHQ1, positioned at the ends of the methylated oligonucleotide so as to form a FRET (Förster resonance energy transfer) pair, is sensitive to fluctuations in the conformation of the enzyme–substrate complex. It should be noted that another common approach to detecting conformational dynamics based on measurement of the intrinsic Trp fluorescence of ALKBH3 was unsuccessful due to a low signal-to-noise ratio. The time-dependent FRET signal profiles following rapid (1 ms) mixing of equimolar amounts of enzyme and the m3C_FRET substrate in the presence of Fe(II) and 2OG ligands were obtained under near single-turnover conditions (Figure 3). The data indicate that a specific change in the distance between the dyes, and therefore between the ends of the DNA strand, is only observed for wt and Y143F ALKBH3. In the case of the wt protein, the initial stage of the enzyme–substrate interaction was accompanied by a slight increase in the FRET signal between 20 ms and 2 s. Subsequently, from 10 s to 250 s, a significant quenching of fluorescence occurs, followed by stabilization at a constant level. The kinetic curve obtained for the Y143F mutant was similar to wt ALKBH3, except that the phase of decrease in the signal was shifted towards longer time scales (20–600 s). No reliable changes in the FRET signal were detected in the 1 ms–1000 s time range when mixing the m3C_FRET substrate with the mutant forms Y143A, L177A, and H191A ALKBH3. The absence of a signal decrease phase in the SF curves for the inactive mutants likewise suggests that this transition is associated with the oxidative demethylation step of the process.

Analysis of the reaction product accumulation (Figure 2) indicates that the half-life of the m3C conversion (τ_1/2_) is approximately 60 s for wt ALKBH3 and 240 s for the Y143F mutant, which roughly corresponds to the half-life of the phase of pronounced FRET signal quenching observed on the SF curves (Figure 3). Thus, this conformational transition may be attributed to fluctuations within the catalytically competent complex, resulting in oxidation of the substrate and subsequent release of the repaired product from the active site. Typically, the bending of the dsDNA substrate (spatial approximation of the oligonucleotide ends) upon its accommodation within the active site pocket is accompanied by quenching of the FRET signal. However, in the case of ssDNA oligos, the signal quenching relates to steps of the release of DNA from the enzyme complex. A similar effect was observed on FRET curves during the catalytic demethylation of m1A within ssDNA by the bacterial dioxygenase AlkB, as described in reference [23]. By comparing FRET data for ss and ds substrates, we suggested that the termini of the 15-mer ss m1A substrate are brought together when the oligonucleotide is free in solution and grow apart when it is bound to the enzyme. 

**Figure 4:**
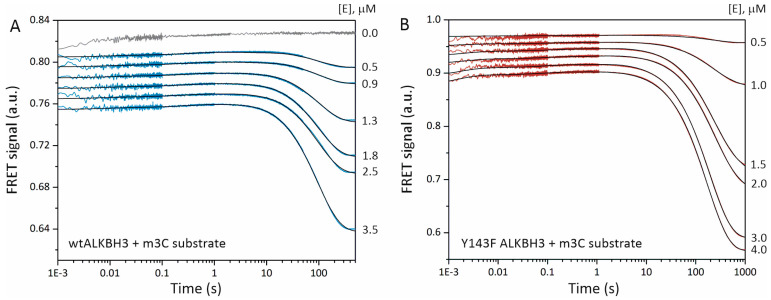
Pre-steady-state kinetics of m3C-containing DNA demethylation by wt (**A**) and Y143F (**B**) ALKBH3 proteins. Time courses of the FRET signal obtained under interactions of 1.5 μM substrate m3C and various concentrations of wt ALKBH3. The gray curve represents a control experiment with no enzyme added. For each set of curves, jagged traces represent experimental data. Smoothed curves were obtained via global fitting to the kinetic Scheme 1.

For wt and Y143F ALKBH3, a series of SF traces were recorded across a range of m3C substrate concentrations from 0.5 to 4.0 µM (Figure 4). The resulting kinetic curves exhibit a similar overall pattern throughout the entire catalytic cycle, suggesting that the protein–DNA complex dynamics are preserved when Tyr143 is substituted with Phe. Additionally, a slight increase in signal, absent in the native protein, is discernible in the initial segment (1–5 ms) of the curves for Y143F ALKBH3, most likely indicative of the primary enzyme–substrate binding. This suggests that a proper orientation of the substrate within the active site of Y143F requires extra conformational adjustment of the m3C substrate structure due to the disruption of Tyr143’s polar interactions within the DNA-binding loop.

**Scheme 1:**
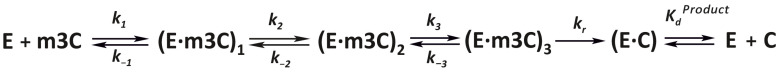
The kinetic mechanism of the enzyme–substrate complex’s conformational transitions in the course of the demethylation process as measured by SF method. E is the enzyme coordinated with Fe(II) and 2OG; m3C is the DNA substrate; C is a repaired cytosine; (E∙m3C)_i_ is an intermediate enzyme–substrate complex. Rate constants *k_i_* and *k_−__i_* (*i* = 1, 2, and 3) characterize forward and reverse directions of the equilibria corresponding to the steps of substrate binding and adjusting for catalysis. Rate constant *k_r_* describes the irreversible step of substrate hydroxylation; *K_d_^Product^* is an equilibrium constant of the enzyme–product complex (E∙C).

The amplitude of the FRET signal fluctuations exhibits a pronounced concentration dependency, enabling the approximation of the experimental curves with a system of differential equations using the DynaFit 4 software [24] and describing them with a kinetic scheme (Scheme 1). It is noteworthy that during the fitting of the molecular-kinetic mechanism, schemes with fewer than three intermediate complexes (E·m3C)_i_ resulted in a low level of correlation between the theoretical and experimental curves. Table 1 summarizes the values of the rate constants describing the forward and reverse reactions of the mechanism, as well as the equilibrium dissociation constant of the enzyme–product complex. As inferred from the constant *k*_1_ values, the formation of the primary enzyme–substrate complex (E·m3C)_1_ with the Y143F mutant (*k*_1_*^Y143F^*= 19 × 10^6^, M^−1^ s^−1^) leads to the substrate’s conformational change arising earlier than under interaction with wt ALKBH3 (*k*_1_*^wt^* = 94 × 10^6^, M^−1^ s^−1^). Subsequent isomerization of the enzyme–substrate complex is characterized by two equilibrium steps, which occur at half the rate in wt ALKBH3, yet with greater efficiency than for the Y143F protein. To assess the overall affinity of ALKBH3’s active site for damaged DNA, the equilibrium constant *K_a_* = *K*_1_ + *K*_1_ *× K*_2_ + *K*_1_ *× K*_2_ *× K*_3_, where *K_i_* = *k_i_/k_−i_*, was determined. The *K_a_* values for the wt and Y143F proteins are very close, indicating that the efficiency of pre-catalytic steps is independent of the presence of the Tyr’s OH moiety at position 143. In contrast, the rate of the step of irreversible hydroxylation of the methyl group by the native protein (*k_r_^wt^* = 0.023 s^−1^) is nearly twice as fast as that of the Y143F mutant (*k_r_^Y143F^* = 0.013 s^−1^). Furthermore, the equilibrium dissociation constant of the enzyme–product complex (*K_d_^Product^*) is almost an order of magnitude higher for wt ALKBH3. Thus, we suggest that the Y143F ALKBH3-DNA product complex exhibits increased stability, and the release of the reaction product from the active site may be impeded. The substitution of Tyr143 with Phe leads to a reduction in the rate and efficiency of demethylation of the m3C substrate, likely due to perturbations in the structure of the catalytically competent complex and strong product binding, which complicates the enzyme’s transition to the next turnover.

### 2.3. Equilibrium Binding of ALKBH3 to Metal Ions, Co-Substrate, and Methylated DNA

To investigate the impact of selected mutations on ALKBH3’s ability to bind its cofactor, co-substrate, and methylated DNA, the fluorescent titration method was employed. It is well-established that enzyme–ligand binding typically leads to a protein conformational change, which in turn can alter the fluorescence intensity of tryptophan and tyrosine residues within the protein sequence. Previous studies have demonstrated that the addition of various ligands to dioxygenases of the AlkB family is accompanied by the quenching of its fluorescence [7,22,23]. In this study, transitional metals (Fe(II), Ni(II), and Co(II)), 2OG, and the m3C substrate served as titrants. Protein fluorescence quenching was measured after the addition of an excess of the ligand to 1 µM of enzyme (Supplementary Figure S2). To determine the equilibrium dissociation constant’s *K_d_* values, the resulting titration curves were analyzed using a non-linear regression algorithm (see Section 4.3 of “Materials and Methods” for details). Previous investigations of the binding capacity of AlkB and ALKBH2 dioxygenases have established that *K_d_* values for complexes with transitional metals and 2OG are in the range of 15–50 µM, and of 0.5–3 µM for methylated DNA [7,25]. As indicated by the data presented in Table 2, the stability of the wt ALKBH3 apoenzyme’s affinity to various ligands and damaged DNA generally corresponds to the stability of such complexes for AlkB and ALKBH2 enzymes. Among the metals studied, the Fe(II) ion exhibited the highest affinity for the active site of wt ALKBH3 (*K_d_* = 25 µM). The binding of Ni(II) and Co(II) ions of similar radii was weakened by approximately two-fold. Regarding the binding of wt ALKBH3 with the co-substrate 2OG, the fluorescence titration data indicate a relatively low stability for this complex with a *K_d_* of ~50 µM. It was also revealed that the pre-incubation of ALKBH3 with the Fe(II) or 2OG enhances its ability to form a stable complex with another ligand (2OG or Fe(II)) to a certain degree. This behavior is more characteristic of the bacterial homolog AlkB rather than human ALKBH2 [7].

The introduction of substitutions at selected amino acid residues (Tyr143, Leu177, His191) decreased the affinity for ligands in some cases decrease and enhanced it in other cases (Table 2). Titration of all mutants with Fe(II) ions yielded *K_d_* values similar to those of native ALKBH3, with the exception of the H191A ALKBH3 variant, which formed the most stable complex with iron (*K_d_* = 20 µM). Considering that His191 is involved in the metal coordination, the removal of its side chain was anticipated to weaken the affinity of ALKBH3 to metals, but our data indicate a contrary trend. Further investigation is required to elucidate this phenomenon. Non-native metals such as Ni(II) and Co(II) also bound relatively well to the Y143F, Y143A, and H191A mutants. L177A is the only substitution, which required a larger quantity of Ni(II) and Co(II) to achieve equilibrium, resulting in a significant increase in the *K_d_* up to 90–100 µM. Moreover, the L177A variant exhibited the poorest affinity for 2OG among all the proteins studied. The H191A variant displayed the highest affinity for 2OG among the mutants examined, while L177A exhibited the lowest. The substitution of Tyr143 with Ala weakened the dioxygenase’s ability to bind 2OG by nearly twofold.

The affinity of the active site of ALKBH3 for methylated DNA was studied using the m3C substrate without fluorescent labels (see Section 4). As indicated by the data in Table 2, the *K_d_* value for wt ALKBH3 is 2.8 µM, which is consistent with previously obtained values for the ALKBH2 (*K_d_* = 2.9 µM) and AlkB proteins (*K_d_* = 1.7 µM) [7,25]. The replacement of key amino acids leads to an approximate 1.4-fold weakening of contacts with the DNA substrate for the Y143F mutant, 2.3-fold for Y143A, 2-fold for L177A, and 1.5-fold for H191A. Therefore, the removal of the bulky side chain of Tyr143 dramatically affects the ability of ALKBH3 to bind damaged DNA, while withdrawal of its OH group only slightly weakens protein–DNA interactions. These results are in good agreement with data on the catalytic activity and pre-steady-state kinetics of the proteins described in Section 2.1 and Section 2.2 Based on the data obtained, it can be concluded that the deletion of the side chains of Tyr143 and Leu177 introduces significant distortion into the active site pocket and perhaps the whole ALKBH3 globule, influencing the enzyme’s ability to form specific complexes with ligands and DNA.

### 2.4. Circular Dichroism for Protein Folding Analysis

**Figure 5:**
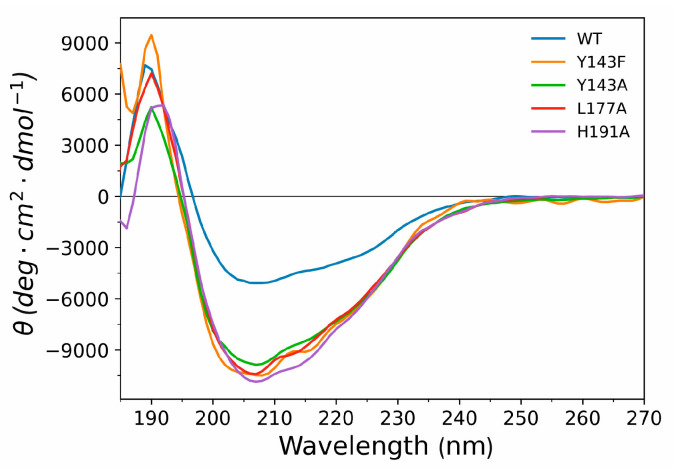
Far-UV CD spectra of wt (blue), Y143F (yellow), Y143A (green), L177A (red), and H191A (purple) ALKBH3 collected from 0.2 mg/mL protein at 11 °C in the absence of ligands. Each spectrum was measured three times, corrected for background, averaged, and expressed as mean residue ellipticity θ (deg·cm^2^·dmol^−1^).

To assess the effect of key amino acid substitution on the ALKBH3’s secondary structure content circular CD spectra of each protein variant in the apo state were recorded in accordance with experimental conditions optimized in our previous study [26]. The spectrum of wt ALKBH3 exhibited a positive peak at 195 nm and two negative peaks at 208 and 218 nm, indicating the presence of α-helical and β-strand content (Figure 5). Overall, the spectrum displayed a high degree of similarity with the spectrum of the bacterial dioxygenase AlkB [21]. In the spectra of all studied mutants, we observed a pronounced increase in intensity between 200 nm and 230 nm, suggesting possible alterations in the secondary structure content as compared to the wt protein.

The secondary structure content was determined by processing experimental spectra using the DSSP (BeStSel, Budapest, Hungary) [27] and CDSSTR (DichroWeb, London, UK) [28] algorithms. The results of the calculations, performed by different methods, showed a good correlation with each other, with the divergence of theoretical curves from experimental data in terms of normalized root mean square deviation (NRMSD) not exceeding 0.048. A detailed description of the parameter optimization process for each algorithm is provided in our recent work [26]. The BeStSel package was selected for further analysis as it provides more information regarding the structural organization of the protein. As follows from data summarized in Table 3, antiparallel β-strands predominate in the structure of the wild-type ALKBH3 (37.6%), with a considerable portion existing in right-twisted and relaxed conformations. The protein sequence also contains a significant number of loop motifs (12.6%) and poorly ordered structures (~44.7%), which may constitute the N-terminal domain. Only 5.2% of the amino acid sequence is composed of α-helices. Utilizing the protein architecture and topology prediction feature available in BeStSel, it was established that the obtained CD spectrum belongs (with a probability of 96%) to the class of β-proteins with a sandwich or β-barrel architecture. Such a distribution corresponds to the structure of ALKBH3 determined by X-ray crystallography for a truncated form of the enzyme lacking 69 amino acids from the N-terminus (PDB 2IUW) [14]. Specifically, the β7-β14 strands form two antiparallel sheets of a central “jellyroll” fold, which constitutes the catalytically active DSBH domain (Figure 1). Thus, the CD method we selected enabled us to determine the composition of the secondary structure of ALKBH3 with a high degree of precision.

The analysis of CD spectra of ALKBH3 mutants indicates that even a single amino acid substitution leads to significant rearrangements in the protein structure. For instance, the substitution of Tyr143, located on the edge of the DNA-binding pocket, with the more hydrophobic Phe results in an almost twofold increase in the α-helix content, which apparently occurs at the expense of a reduction in the antiparallel β-strands (Table 3). The complete removal of the bulky amino acid in the Y143A mutant leads to further decrease in the proportion and diversity of β-strands, and the number of α-helices and loop motifs increases over wt ALKBH3 by 3- and 1.5-fold, respectively. Topological analysis shows that while the Y143F mutant can still be classified as a β-protein with a probability of 63.4% exhibiting a “β-barrel” or “jelly roll” architecture, the structure of Y143A ALKBH3 already belongs with an 85% probability to the class of αβ-proteins with a 2-layer “sandwich” architecture.

The substitution of Leu177, situated deep inside the active site pocket as part of the β7 strand, with Ala leads to an increase by 2.7-fold in the α-helical content on the one hand, and a disappearance of left-twisted β-strands on the other. Right-twisted β-strands of L177A ALKBH3 are maintained in a quantity comparable to the wt protein, but relaxed β-strands (which are actually slightly right-twisted) are reduced by threefold. The side chain of Leu177 was found to be a mixture of the normal and oxidized forms, with only the oxidized form being capable of coordinating 2OG via an H_2_O molecule [14]. This network of hydrogen bonds further extends to Arg275, as well as the Fe(II) ion. It is conceivable that the removal of the Leu177 side chain could perturb the structure of the β-sheet to which it belongs, and also potentially alter the secondary structure of the opposing motifs involved in hydrogen bonding to Leu177. Topologically, L177A ALKBH3 more closely resembles an αβ-protein (70%) with a “roll” architecture, rather than a β-protein (28%).

His191 is located at the C-terminus of a β8 strand and directly participates in the Fe(II) ion coordination. The secondary structure composition of the H191A mutant indicated a substantial increase in the amount of helical elements (up to 3.1-fold) and a slight increase in the turn content (by 4%). On the other hand, the amount of relaxed antiparallel β-strands decreases by 9.5-fold, while a small number of parallel β-strands (1.1%) begin to appear. Given the proximity of His191 to the loop motif, the removal of its bulky positively charged side chain may induce rearrangements in the region of the residue itself. Topological analysis revealed a high degree of similarity in architecture between the H191A and Y143A mutants.

It should be noted that for all mutants, we observed a rise in the content of poorly ordered elements over wt ALKBH3 by 1.5–5%. This effect could be attributed to an increased amount of noncanonical structures such as π-helix, β-bridge, and the like.

### 2.5. Thermal Stability of ALKBH3

**Figure 6:**
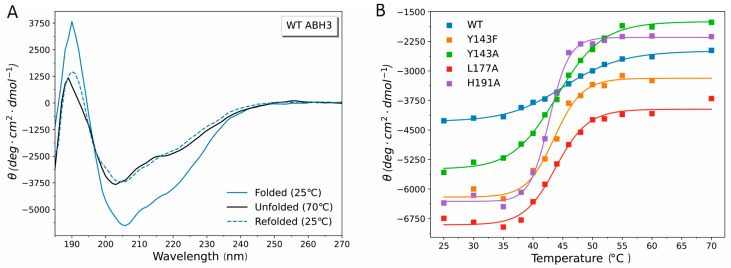
Thermal stability of wt and mutant ALKBH3 proteins measured by CD spectroscopy. (**A**) Spectra of wt ALKBH3 between 180 and 270 nm at 25 °C (light blue), after heating to 70 °C (dark blue), and cooling again to 25 °C (dashed light blue). (**B**) Representative melting profiles of wt (blue), Y143F (yellow), Y143A (green), L177A (red), and H191A ALKBH3 (violet) derived from the θ values at 218 nm. The unfolding transitions were monitored 0.2 mg/mL of the apoprotein in 10 mM Potassium Phosphate (pH 7.8) and 50 mM (NH_4_)_2_SO_4_.

The effect of substitutions of conserved amino acids in the active site of ALKBH3 on its thermal stability was studied by circular dichroism (CD) spectroscopy combined with thermal denaturation of the samples. The CD data were collected for wt and mutant apoproteins at various temperatures (25–70 °C) with 2–5 °C intervals (Supplementary Figure S3). It was found that ALKBH3 undergoes irreversible denaturation upon heating, as the spectrum did not regain its shape and amplitude after a cycle of heating-cooling (Figure 6A). However, even after reaching the maximum degree of denaturation, the spectrum partially retains characteristic elements, suggesting that the unfolded ALKBH3 protein is in a molten globule state [29]. Melting profiles expressed as a temperature dependence of the mean residue ellipticity at 218 nm were largely sigmodal, indicating the presence of a single transition between the folded and unfolded state (Figure 6B). 

The melting profiles were analyzed quantitatively using characteristic wavelengths between 208 and 222 nm by a single-stage model [30]. 

Upon fitting, the thermodynamic parameters (*T_m_*, Δ*H*, Δ*S*, and Δ*G*) were determined (Table 4, Supplementary Figure S4). The highest thermal stability was observed for wt ALKBH3 with a *T_m_* of 45.5 °C and an unfolding enthalpy of 42 kcal/mol. The substitution of conserved amino acids resulted in a decrease in thermal stability, as evidenced by lower *T_m_* values, and an increase in both enthalpy and entropy values. Specifically, the thermal unfolding profiles of Y143F ALKBH3 yielded a reduction in melting temperature by 2.1 °C and an increase in Δ*H* and Δ*S* values by 32 kcal/mol and 103 cal/mol·K, respectively. The thermodynamic parameters of the Y143A mutant had values closer to those of native ALKBH3. Given that the Y143F mutant retains its demethylase activity, it can be hypothesized that the structural rearrangements prompted by the removal of the polar OH moiety in the DNA-binding loop region may facilitate a loosening of the globule, thereby enhancing the unfolding entropy. The absence of the bulky Tyr143 side chain has a marginal effect on the Y143A mutant’s thermal stability and cooperativity of denaturation, yet it almost completely abolishes the demethylase activity of ALKBH3. The unfolding thermodynamics of the L177A mutant largely parallel those of the Y143F mutant, with a considerable increase in Δ*H* and Δ*S* parameters and a slight decrease in *T_m_* by 1.1 °C relative to the wt protein. A distinctive feature of the H191A mutant is that it exhibits the lowest thermal stability (*T_m_* = 42.8 °C) and highest unfolding enthalpy and entropy (Δ*H*_H191A_ = 120 kcal/mol, Δ*S* = 403 cal/mol·K) in comparison to other ALKBH3 forms. In addition, H191A ALKBH3 has the highest unfolding free energy among the studied proteins. The large contribution of the entropic component to Δ*G* value (Figure S4) is probably explained by the presence of extra H bonds and hydrophobic interactions in the structure of this mutant than in the wt ALKBH3. Taking into account that the packing density of the ALKBH3 globule is close to 0.76 (PDB ID 2IUW), we can expect the denaturation to occur in a phase change manner and in a narrow temperature range. Thus, the number of hydrogen bonds formed during transition into a polar solvent is a measure of the protein’s stability.

## 3. Discussion

The plasticity of the structure of the AlkB family of dioxygenases is a paramount factor in determining their broad substrate specificity. As evidenced by numerous studies employing biochemical, biophysical, and computational approaches, the conformational mobility of not only specific loop motifs (i.e., strand determination hand and nucleotide recognition lid, NRL) but also the β-sheet catalytic core, and the damaged DNA strand are essential for the selectivity of DNA dioxygenases [18,19,21,22,31,32]. In previous works, we have shown that *E. coli* AlkB and human ALKBH2 dioxygenases undergo a cascade of conformational transitions that change sequentially along the reaction coordinate [7,23]. Moreover, an alkylated DNA substrate was also found to be susceptible to dynamic conformational changes, with the pattern of transitions depending on whether the substrate was single- or double-stranded. With regard to the human ALKBH3 dioxygenase, no similar studies had been conducted before. This work represents the first attempt to characterize the structural dynamics of the ALKBH3-DNA complex in solution during a single enzyme turnover. We also explored the impact of substituting conserved amino acids Tyr143, Leu177, and His191 on the protein’s activity, structure, and dynamics. A comprehensive approach was developed, utilizing circular dichroism (CD) spectroscopy, stopped-flow (SF) kinetics, equilibrium fluorescence titration, and site-directed mutagenesis.

By ultrafast mixing (at a time point of approximately 1 ms) of the enzyme with damaged ssDNA in a cuvette of the SF spectrophotometer, we examined the conformational dynamics of the ALKBH3-DNA complex accompanying the demethylation process. It was determined that the m3C substrate undergoes at least three conformational transitions on the way to a catalytically competent state under interactions with the native protein. The repair process was described by a five-step kinetic mechanism that encompasses the steps of the binding of damaged DNA by ALKBH3, specific isomerization of the enzyme–substrate complex, and formation of the demethylated reaction product. Surprisingly, the trend of FRET signal change observed for ALKBH3 closely resembles the one previously generated during the study of the conformational dynamics of the AlkB-ssDNA complex (see Figure 3 in ref. [23]). Furthermore, the conformational transitions in AlkB-ssDNA and ALKBH3-ssDNA complexes, as monitored by FRET, are described by the same kinetic scheme. This implies that the ALKBH3 dioxygenase shares more similarity with AlkB than with ALKBH2 in terms of conformational dynamics. By comparing the kinetic parameters obtained for ALKBH3 (Table 1) and AlkB (Table 2 in ref. [23]) under pre-steady-state conditions, we can conclude that the initial binding of the enzyme to its substrate and further stabilization of the damaged base in the active site proceed at nearly identical rates and with comparable efficiency (in terms of *k*_1_, *k*_−1_, *k*_2_, *k*_−2_, *k*_3_, and *k*_−3_ values). The subsequent hydroxylation of the substrate’s methyl group involving the Fe(IV)-oxo species occurs significantly more slowly in the case of ALKBH3 (*k_r_^ALKBH3^* = 0.023 s^−1^, *k_r_^AlkB^* = 0.19 s^−1^). Similar patterns in the *k_cat_* values have been identified under investigation of the steady-state kinetics of these DNA dioxygenases [33]. Apparently, the bacterial dioxygenase operates faster and more efficiently than its human homolog, primarily due to the high rate of the catalytic step and product release. 

**Figure 7:**
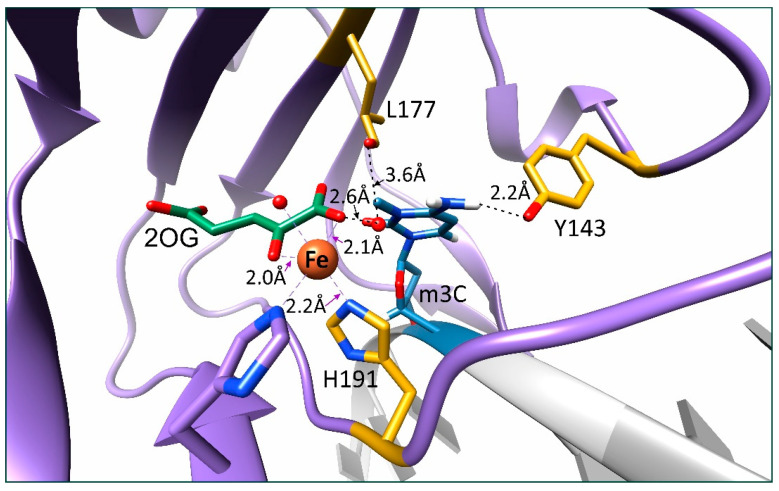
Close-up view of the active site of ALKBH3 in the presence of bound m3C-containing ssDNA. The structure of the ALKBH3-DNA complex with the Fe(II) and 2OG bound was created by overlaying the crystal structure of 2IUW [14] and a 15nt m3C substrate using the UCSF Chimera software (San Francisco, CA, USA). The completed structure can be found in the Mendeley Data repository (http://dx.doi.org/10.17632/7bfsjtkgtb.1, accessed on 7 December 2022). The protein and nucleic acid chains are shown in purple and grey, respectively. Residues selected for mutation are colored yellow. The m3C coordinated in the catalytic center is blue.

According to X-ray data, Tyr143 is located in an extended loop motif between α2 and β6, which is not highly conserved (with the exception of Tyr141, Tyr143, Ser144, and Ile146) (Figure 7). The structure-based alignment has shown that the loop of ALKBH3 contains a small helical segment adjacent to Tyr143, whereas in the structure of AlkB, two strands, β3 and β4, lie in the same position [14]. In addition, this loop region in ALKBH3 is significantly shorter compared to the corresponding region in AlkB. Nonetheless, it is hypothesized that this motif in the ALKBH3 protein is critical for DNA substrate coordination, as it opposes the NRL motif [14,34]. Indeed, the superimposed structures of ALKBH3 and a 15-mer substrate m3C indicate a possible H-bonding between the NH_2_ of cytosine and the OH moiety of Tyr143, which is directed into the active site cleft (Figure 7). Replacing Tyr143 with Phe or Ala allowed for the independent identification of the role of the polar OH moiety and the entire side chain of this amino acid. The findings suggest that the presence of a bulky hydrophobic side chain at position 143 is essential for ALKBH3 activity, while the removal of the hydroxyl retains up to 30% of the protein’s demethylating capacity. The Y143F substitution slightly decreases the apo-enzyme’s affinity for damaged DNA (as shown by fluorescence titration analysis), but does not affect the holo-enzyme’s ability to bind the m3C substrate (according to the SF analysis). Monitoring the conformational dynamics of the enzyme–substrate complex revealed an additional step of bending of the DNA substrate bound to the Y143F mutant upon binding, compared to wt ALKBH3. However, this does not impact the efficiency and rate of the pre-catalytic steps. At the same time, the release of the demethylated product from the Y143F mutant can be complicated because the stability of the enzyme–product complex of Y143F ALKBH3 significantly exceeds that of wt ALKBH3. Thus, in the absence of Tyr143’s hydroxyl moiety, the repair process is likely slowed down at the step of the catalytic hydroxylation of m3C, and the demethylation product remains tightly bound to the active site. Based on the data, we conclude that the disruption of polar interactions between the m3C base and Tyr143 slightly affects the transformation of the substrate anchored in the active site, whereas the removal of the bulky side chain at position 143 significantly impedes the efficient fixation of the damaged base due to the disruption of the network of hydrophobic interactions.

**Figure 8:**
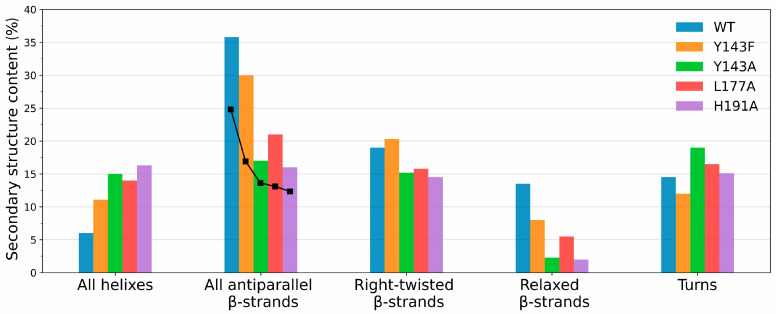
Representation (in percent) of certain types of secondary structures for wt ALKBH3 and its mutants. The diagram is built using the data in Table 3. Black dots represent the comparative level of demethylation activity after 40 min of incubation in a standard assay (see Section 2.1 for details). Each dot corresponds to the protein on whose column it is placed.

There is a little research of the structure and thermodynamics of AlkB-like dioxygenases in solution. The folding and dynamics of *E. coli* AlkB upon binding to Fe(II), 2OG, and succinate were analyzed by the combination of NMR, fluorescence, and CD spectroscopy [21,22,35]. Here we employed circular dichroism spectroscopy for the biophysical characterization of the ALKBH3 protein. The results show that wt ALKBH3 possesses a relatively flexible structure with a high content of antiparallel β-sheets, loop regions, and disordered motifs. Thermal denaturation of the native protein is characterized by a single broad transition and a lower *T_m_* value (45.6 °C) compared to AlkB (54 °C) [21]. Mutation at key residues (Tyr143, Leu177, and His191) resulted in a significant distortion of the globular structure and a decrease in protein thermal stability. As demonstrated by a correlation diagram (Figure 8), the structure of inactive mutants with Ala substitutions manifests an increase in the amount of various types of α-helices, while the presence of relaxed and right-twisted β-strands decreases significantly. In other words, the weakening of the demethylating activity of ALKBH3 (black dots on the histogram columns) positively correlates with the reduction in the β-strand component. We assume that the DNA binding loop containing Tyr143 may extend its short helical component as a result of Tyr-to-Ala substitution. 

Further studies should verify this hypothesis. Interestingly, a proportion of secondary structure components of the partially active Y143F mutant occupies an intermediate position between the wt ALKBH3 and inactive mutants. At the same time, Y143F is thermodynamically less stable than the inactive Y143A mutant.

Overall, the distortion induced by the removal of Tyr143, Leu177, and His191 side chains contributes to a reduction in the stability of the ALKBH3 globule due to an increase in the entropy of unfolding. In the case of inactive mutants, the denaturation process was accompanied by a sharp and rapid transition, indicative of the presence of a large number of cooperative units inside the protein domains. It seems the Ala substitution of the ALKBH3 amino acids involved in coordinating the specific ligand (i.e., cofactor or co-substrate) does not necessarily disrupt the protein affinity to that ligand. In particular, we have found that the H191A mutant, lacking the His which belongs to the iron-coordinating triad, unexpectedly retains the ability to form a complex with transition metals at the level of the wt enzyme. Thus, the lack of activity by H191A ALKBH3 is rather due to the disruption of the octahedral coordination arrangement of iron, which prevents the accommodation of the O_2_ molecule and the subsequent decarboxylation of 2OG.

## 4. Materials and Methods

### 4.1. Protein Expression and Purification

Plasmids based on the pET28a vector encoding Y143F and Y143A variants of ALKBH3 with the N-terminal His-tag were obtained by site-directed mutagenesis according to standard QuickChange protocol (La Jolla, CA, USA). Plasmids encoding variants L177A and H191A were purchased from Evrogen Company (Moscow, Russia). The presence of correct mutations was verified by the sequencing of the entire coding sequence.

The wt and mutant ALKBH3 proteins were overexpressed in *E. coli* Arctic Express cells in LB medium containing 50 µg/mL of kanamycin at 15 °C and purified by a universal protocol. The cells were harvested, lysed in the presence of a protease inhibitor cocktail (EDTA-free, Abcam, Cambridge, UK), and the lysate was centrifuged for 30 min at 30,000× *g*. The supernatant was loaded onto a column containing a Q-Sepharose Fast Flow resin (Cytiva, Uppsala, Sweden) equilibrated with 20 mM of HEPES-KOH (pH 7.8) and 40 mM of NaCl. The flow-through fraction was collected, adjusted to 300 mM of NaCl, and then applied to a 1 mL HiTrap Ni-Chelating column (Cytiva, Uppsala, Sweden). The protein of interest was eluted with a 10 mL linear gradient of 0 to 500 mM of imidazole. The purified protein was supplemented with glycerol (up to 50%) and DTT (up to 2 mM) and stored at −20 °C. The concentration of proteins was determined by the Bradford assay. The protein purity was verified using SDS-PAGE.

### 4.2. Oligonucleotides

Oligodeoxyribonucleotides (15mer, Table 5) containing normal bases, methylated cytosine (m3C), and 6-carboxyfluorescein (6-FAM) were synthesized on an ASM-700 Synthesizer (BIOSSET Ltd., Novosibirsk, Russia) using phosphoramidites purchased from Glen Research (Sterling, VA, USA) and ChemGenes (Wilmington, MA, USA). The purification procedure included an anion exchange HPLC method. The concentration of oligonucleotides was determined by measuring UV absorbance at 260 nm. The extinction coefficient (ε) of m3C base was calculated as its unmodified counterpart. The purity, homogeneity, and integrity of each oligonucleotide were assessed by 20% denaturating PAGE. 

### 4.3. Enzymatic Reaction

An enzymatic hydroxylation of a single-strand m3C-containing DNA was studied using a chemical quenching technique recently described for the ALKBH2 dioxygenase in [7]. Briefly, a typical reaction mixture contained an equimolar amount (2 µM) of the protein and FAM-labeled DNA substrate (m3C_FAM) in a buffer consisting of 50 mM of HEPES-KOH (pH 7.8), 50 mM of KCl, 10 mM of MgCl_2_, 1 mM of 2OG, 2 mM of sodium ascorbate, and 40 µM of (NH_4_)_2_Fe(SO_4_)_2_·6H_2_O. An equal volume of 0.2 M NaOH was added to 5 µm of aliquots of the mixture to terminate the reaction at certain time points. After neutralization with HCl and desalting, each of the aliquots was supplemented by the complementary DNA strand and treated with the HpaII restriction endonuclease specific to the repaired sequence (CCGG) according to the manufacturer’s protocol. The resulting reaction product was separated from the substrate using denaturating PAGE and visualized by the Amersham Typhoon Imager (Cytiva, Uppsala, Sweden) (Supplementary Figure S1). Data for the wt, Y143F, and Y143A proteins were converted to mol/L units and then fitted to a single exponent (Equation (1)), where *P*, *P*_0_, and *P_max_* are amounts of product at any given time, at the zero-time point, and the end time point, respectively; *k_obs_* is the observed rate constant.

(1) $$P=P_{0}+P_{max}(1-\exp\left(-k_{obs}t\right))$$

### 4.4. Equilibrium Fluorescence Spectroscopy

The binding affinity of enzymes to damaged DNA and ligands was measured by equilibrium fluorescence titration on a Cary Eclipse fluorimeter (Agilent, CA, USA) in a cuvette equilibrated at 25 °C. The intrinsic tryptophan fluorescence quenching was monitored at the maximum of emission (345 nm, in most cases) using an excitation wavelength of 280 nm, and 5 nm slits. An amount of 2 mM of (NH_4_)_2_Fe(SO_4_)_2_·6H_2_O, 2 mM of NiSO_4_×7H_2_O, 2 mM of CoSO_4_·7H_2_O, 2 mM of 2OG, or 100 µM of m3C substrate was titrated into 1 µM of apoprotein (a free enzyme with no metal or 2OG added) in the presence of 50 mM of HEPES-KOH (pH 7.8), 50 mM of KCl, and 10 mM of MgCl_2_. When titrating with DNA, the buffer was supplemented with 2 mM of EDTA. The dissociation constant *K_d_* for a model of one-step binding (*E* + *L* ↔ *EL*) was determined using Equations (2)–(5) and a non-linear regression algorithm in the OriginPro 8.1 software (OriginLab Corp., Northampton, MA, USA):

(2) $$K_{d}=\frac{\left[E\right][L]}{\left[EL\right]}$$

(3) $$F=f_{0}+f_{1}\left[E\right]+f_{2}[EL]$$

(4) $$e_{0}=\left[E\right]+[EL]$$

(5) $$l_{0}=\left[L\right]+[EL]$$

where *F* is the observed fluorescence intensity at any given ligand (*L*) concentration; *f*_0_ is the background fluorescence; *f*_1_ and *f*_2_ are partial fluorescence intensities of the free enzyme (*E*) and enzyme–ligand complex (*EL*), respectively; *e*_0_ and *l*_0_ are total concentrations of the enzyme and ligand.

### 4.5. CD Spectroscopy

Far-UV CD spectra from 185 to 280 nm were recorded using a J600 spectrometer (Jasco, Tokyo, Japan) in a 0.1 cm path lengths quartz cuvette (Starna Cells, CA, USA). In all cases, the protein sample (~50 nmol) was dialyzed two times against the CD buffer containing 10 mM of Potassium Phosphate (pH 7.8) and 50 mM of (NH_4_)_2_SO_4_. The protein concentration was verified by absorption at 280 nm and adjusted to 0.2 mg/mL. CD spectra for the determination of secondary structures were collected three times (each spectrum was an average of 10 repeats) at a speed of 50 nm/min and a 1 nm step size (spectral bandwight 2 nm). To increase stability, the protein samples were cooled 11 °C during recording. Data were corrected for a baseline by subtraction of the buffer spectrum and expressed as mean residue ellipticity [θ] (deg.·cm^2^·dmol^−1^). To estimate the secondary structures, each spectrum between 180 and 250 nm was analyzed by both the DichroWeb [28] and BeStSel [27] online programs using the CDSSTR method.

Thermal stability experiments were performed under the same conditions. The spectra were collected from 25 to 70 °C, with the samples incubated at each temperature for 3–5 min before measuring. After that, each sample was gradually cooled to 25 °C and the spectrum was recorded again to observe possible refolding of the protein. Melting curves were corrected for background, transformed to the mean residue ellipticity units, and smoothed using the Savitzky–Golay method with a polynomial order of 3. Values of [θ] at wavelengths of 208 and 218 nm as a function of temperature were used to determine the thermodynamics of folding [30]. The values of Tm and ΔH were determined by non-linear regression analysis with the help of Equations (6)–(8):

(6) $$\theta_{t}=\theta_{u}+\left(\frac{\left(\theta_{f}-\theta_{u}\right)K}{1+K}\right),$$

(7) $$K=\exp\left(\left(\frac{\Delta H}{1.987\cdot t}\right)\left(\frac{t}{T_{m}}-1\right))=exp(-\frac{\Delta H-t\cdot \Delta S}{1.987\cdot t}\right),$$

(8) ΔG=−RTlnK

where *θ_t_*—the mean residue ellipticity (in deg·cm^2^·dmol^−1^) at any given temperature *t*; *θ_u_*—the mean residue ellipticity of the unfolded protein; *θ_f_*—the mean residue ellipticity of the folded protein; K—the constant of unfolding; *T_m_*—the melting temperature; Δ*H*—the enthalpy of unfolding, Δ*S*—the entropy of unfolding.

## 5. Conclusions

In summary, the present study investigated the effects on the protein structure, dynamics, and the DNA demethylation process of substituting key conserved residues in ALKBH3. The results show that the removal of the side chains of Tyr 143, Leu177, and His191 induces profound alterations in the secondary structure of the protein. We suggest that Tyr143 serves as one of the key participants in m3C-containing DNA coordination. Specifically, the polar hydroxyl moiety of this residue facilitates the reaction product’s dissociation from the enzyme’s active site by means of its interaction with the amino group of m3C. The bulky hydrophobic side chain of Tyr143 is important for anchoring and holding the damaged base within the active site pocket. Leu177 appears to play a significant role in sustaining the overall geometry of the DNA-binding cleft. The absence of the His191 side chain does not influence the metal-binding ability of ALKBH3, but impedes the subsequent activation of the Fe(II) ion. The combination of biochemical and biophysical approaches has clearly demonstrated the dynamic nature of the ALKBH3-DNA complex. The results of SF analysis further underscore a close homology between human ALKBH3 and *E. coli* AlkB with regard to structural and dynamic peculiarities of the catalytic process.

## Figures and Tables

**Table 1:** Kinetic parameters of wt and Y143F ALKBH3 activity on the m3C-containing DNA substrate derived from the global fitting of SF data to Scheme 1.

	wt ALKBH3	Y143F ALKBH3
*k*_1_ × 10*^−^*^6^, M^−1^ s^−1^	19 ± 3	94 ± 3
*k*_−1_, s^−1^	64 ± 5	270 ± 26
*k*_2_, s^−1^	2.9 ± 0.9	6.1 ± 1.4
*k*_−2_, s^−1^	2.8 ± 0.5	7.7 ± 0.24
*k*_3_, s^−1^	0.57 ± 0.12	1.4 ± 0.1
*k*_−3_, s^−1^	0.13 ± 0.01	0.48 ± 0.03
*k_r_*, s^−1^	0.023 ± 0.003	0.013 ± 0.001
*K_d_^Product^*, M	(7.2 ± 2.1) × 10^−7^	(0.83 ± 0.21) × 10^−7^
*K_a_*, M^−1^	(1.9 ± 0.2) × 10^6^	(1.4 ± 0.1) × 10^6^

**Table 2:** Dissociation constants *K_d_* (µM) obtained by equilibrium titration of ALKBH3 proteins with ligands and DNA at 25 °C.

	Protein	wt	Y143F	Y143A	L177A	H191A
Ligand						
Fe(II)		25 ± 2	23 ± 2	25 ± 3	24 ± 2	20 ± 2
Ni(II)		42 ± 4	42 ± 3	60 ± 7	90 ± 10	50 ± 4
Co(II)		52 ± 4	40 ± 3	44 ± 4	98 ± 9	59 ± 6
2OG		52 ± 7	52 ± 4	53 ± 10	67 ± 8	34 ± 2
m3C substrate		2.8 ± 0.1	3.8 ± 0.5	6.3 ± 0.6	5.7 ± 0.3	4.3 ± 0.3

**Table 3:** Calculated secondary structure content (%) of wild-type and mutant ALKBH3 proteins based on CD spectra deconvolution using the BestSel single-spectrum analysis function.

Protein Name	α-Helixes		Antiparallel β-Strands			Parallel β-Strands	Turns	Other
	Regular	Distorted	Left-Twisted	Relaxed	Right-Twisted			
WT	3.5	1.7	3.4	15.2	19.0	0	12.6	44.7
Y143F	8.2	2.9	1.9	8.8	20.3	0	11.8	46.1
Y143A	9.1	5.9	0	1.4	15.2	0	19.2	49.2
L177A	8.1	6.0	0	5.4	15.8	0	16.5	48.3
H191A	8.4	7.9	0	1.6	14.5	1.1	16.6	49.8

**Table 4:** Thermodynamic parameters of the ALKBH3 protein’s unfolding.

	wt	Y143F	Y143A	L177A	H191A
*T_m_*, °C	45.6 ± 0.7	43.5 ± 0.4	44.2 ± 0.2	44.5 ± 0.6	42.7 ± 0.1
Δ*H*, kcal/mol	43 ± 1	75 ± 5	54 ± 2	79 ± 5	127 ± 9
Δ*S*, cal/mol·K	133 ± 2	236 ± 3	170 ± 0.6	249 ± 1.3	403 ± 0.4
Δ*G*^298^, kcal/mol *	3.4 ± 0.1	4.7 ± 0.3	3.3 ± 0.1	4.8 ± 0.4	6.9 ± 0.5

(*) Calculated from Δ*H* and Δ*S* values reported here at 298 K.

**Table 5:** Single-stranded oligodeoxynucleotide substrates used in this study.

Name	Sequence	Application
m3C	5′-ACAGGATC(m3C)GGCATA-3′	Equilibrium titration
m3C_FAM	5′-(6-FAM)-ACAGGATC(m3C)GGCATA-3′	PAGE analysis of a catalytic activity
m3C_FRET	5′-(6-FAM)-ACAGGATC(m3C)GGCATA-(BHQ1)-3′	Stopped-flow approach

## Data Availability

The data presented in this study are available in the Supplementary Materials section. The completed structure of ALKBH3 with the m3C substrate superimposed can be accessed at the Mendeley Data repository (http://dx.doi.org/10.17632/7bfsjtkgtb.1 (accessed on 7 December 2022)).

## Supplementary Materials

The following supporting information can be downloaded at: https://www.mdpi.com/article/10.3390/ijms25021145/s1.
