# Supplementary material for: The Role of Key Amino Acids of the Human Fe(II)/2OG-Dependent Dioxygenase ALKBH3 in Structural Dynamics and Repair Activity toward Methylated DNA
Source: Int J Mol Sci. 2024 Jan 17;25(2):1145. doi: 10.3390/ijms25021145 (PMC10816986; doi:10.3390/ijms25021145)
Supplement: Supplementary file 1 [file ijms-25-01145-s001.zip › ijms-2775696-supplementary.pdf]

Supplementary Materials

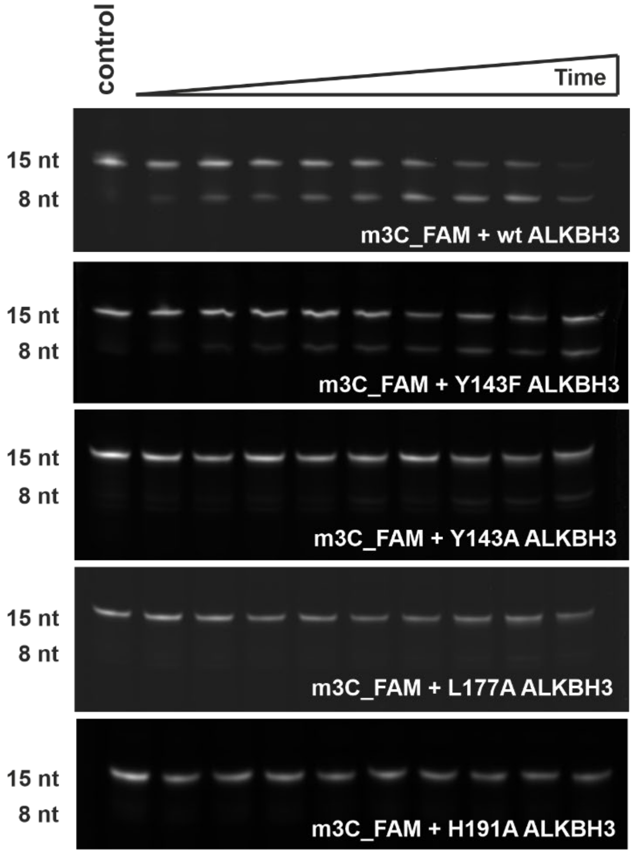

Figure S1. PAGE analysis of ALKBH3 repair activity towards the m3C-containing substrate. ALKBH3 at 2 μM was incubated with an equimolar amount of a FAM-labelled single-stranded substrate m3C_FAM. The reaction was quenched at each time point by the addition of an equal volume of 0.2 M NaOH.

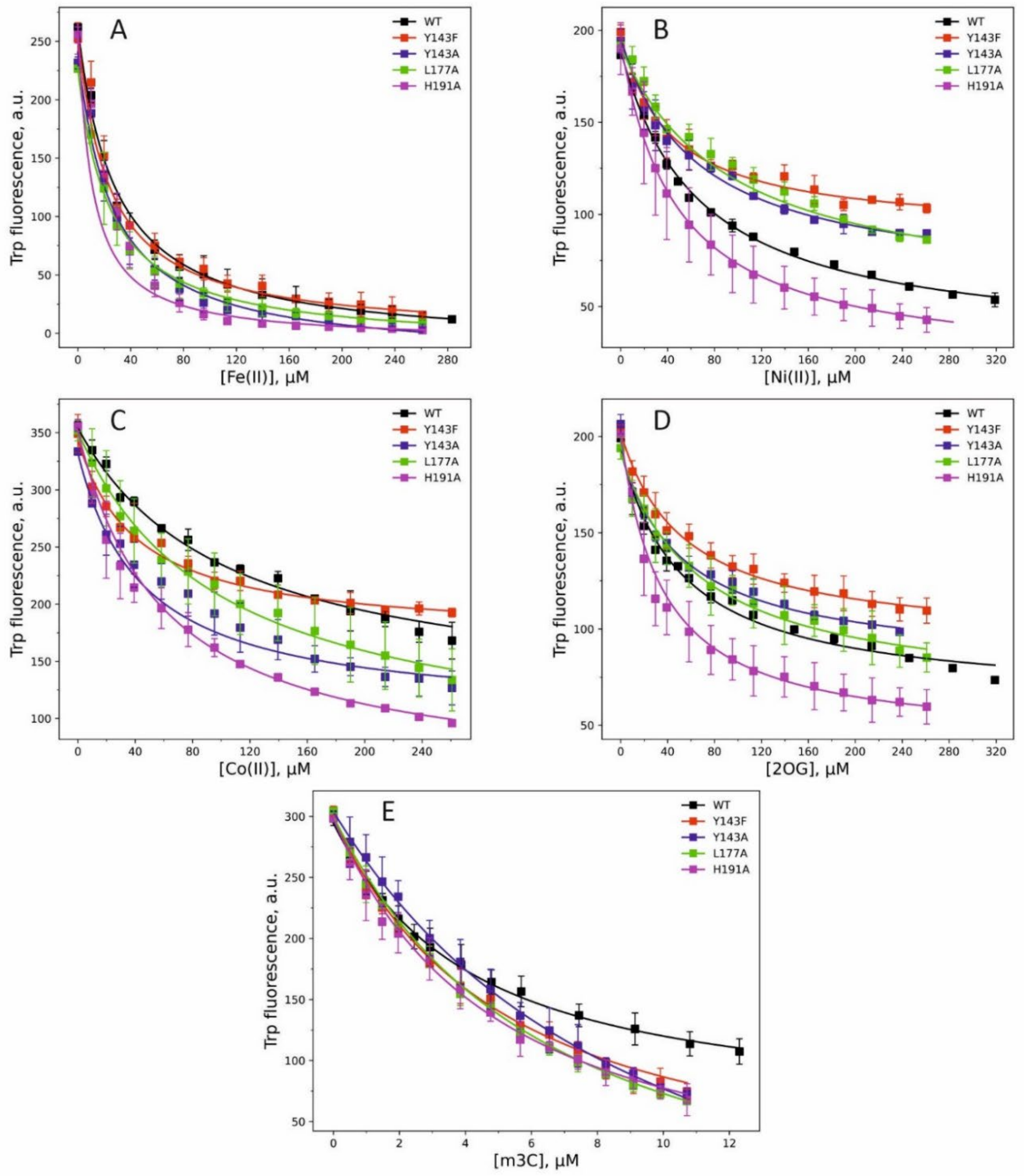

Figure S2. Equilibrium binding of the wild-type and mutant ALKBH3 proteins to transition metals, 2OG, or m3C-containing DNA. The quenching of the enzyme fluorescence is shown as a function of Fe(II) (A), Ni(II) (B), Co(II) (C), 2OG (D), and single-stranded m3C substrate (E). The protein concentration was 1 μM. In the course of titration by Fe(II), a sodium ascorbate (1mM) was added to keep the metal in a reduced state. Experimental data points were fitted to a single-site binding model (see "Materials and Methods"). Each trace represents the average of three technical replicates.

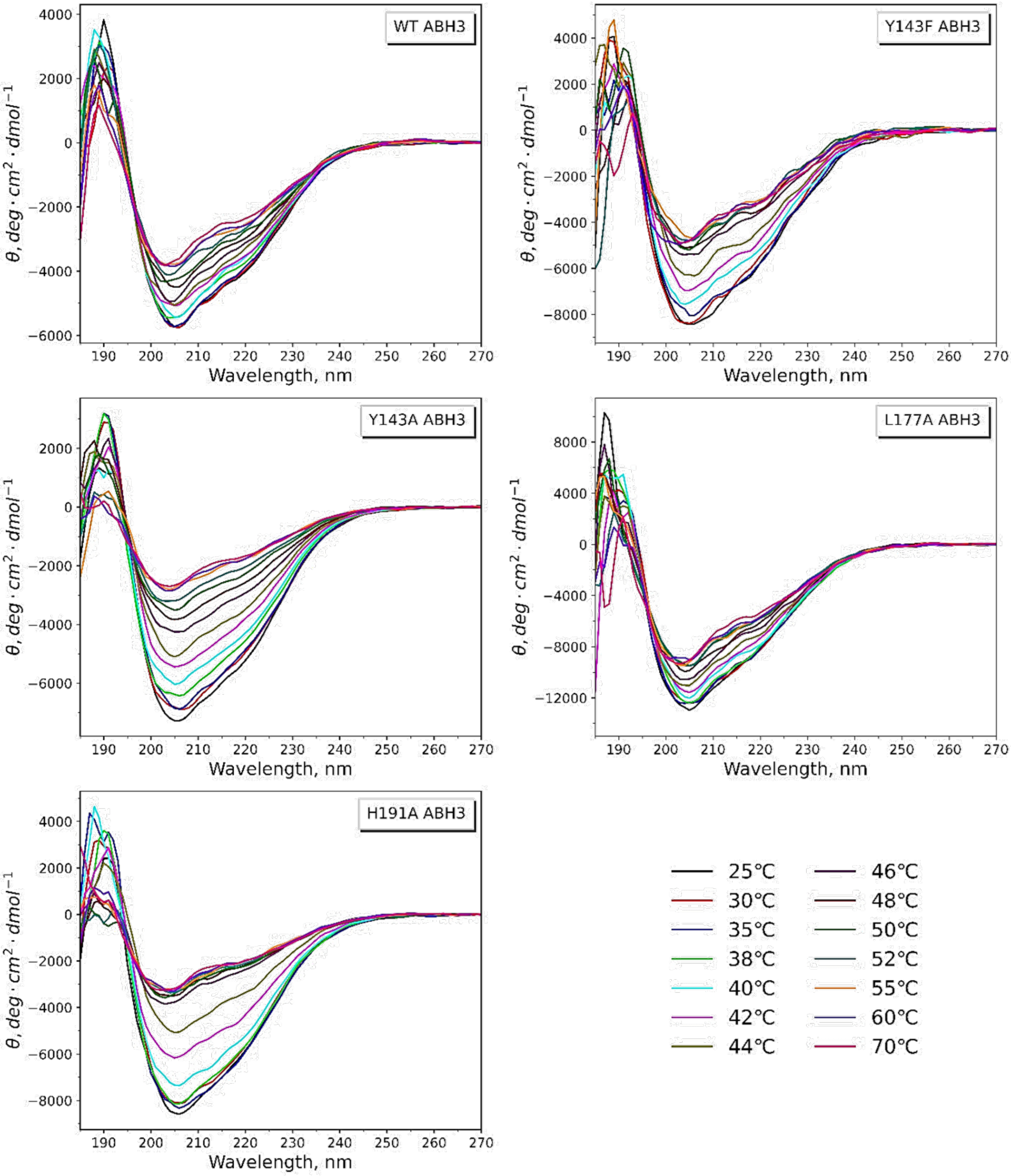

Figure S3. CD spectra of the wt, Y143F, Y143A, L177A, and H191A ALKBH3 proteins scanned at various temperatures and expressed in mean residue ellipticity (θ) units. Spectra were collected in a 0.1 cm cell with 0.16–0.20 mg/ml proteins from 25 to 70°C over a wavelength range of 185 and 270 nm. The data were corrected for background signal and smoothed.

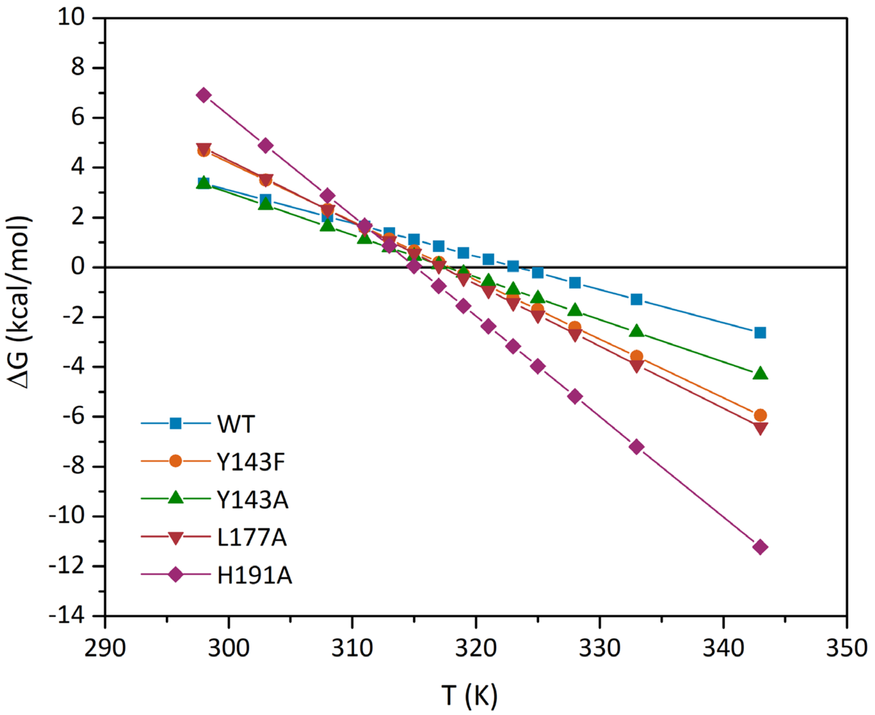

Figure S4. Temperature dependence of free energy (ΔG) of wt, Y143F, Y14.3A, L177A, and H191A ALKBH3 unfolding. The plots were built using the following equation $\Delta G = \Delta H - T^* \Delta S$.
